# Heterologous Production of Microbial Ribosomally Synthesized and Post-translationally Modified Peptides

**DOI:** 10.3389/fmicb.2018.01801

**Published:** 2018-08-07

**Authors:** Yi Zhang, Manyun Chen, Steven D. Bruner, Yousong Ding

**Affiliations:** ^1^Department of Medicinal Chemistry, Center for Natural Products, Drug Discovery and Development, College of Pharmacy, University of Florida, Gainesville, FL, United States; ^2^Department of Chemistry, University of Florida, Gainesville, FL, United States

**Keywords:** RiPPs, heterologous expression, precursor peptide, processing enzymes, synthetic biology, *E. coli*, *Streptomyces*

## Abstract

Ribosomally synthesized and post-translationally modified peptides, or RiPPs, which have mainly isolated from microbes as well as plants and animals, are an ever-expanding group of peptidic natural products with diverse chemical structures and biological activities. They have emerged as a major category of secondary metabolites partly due to a myriad of microbial genome sequencing endeavors and the availability of genome mining software in the past two decades. Heterologous expression of RiPP gene clusters mined from microbial genomes, which are often silent in native producers, in surrogate hosts such as *Escherichia coli* and *Streptomyces* strains can be an effective way to elucidate encoded peptides and produce novel derivatives. Emerging strategies have been developed to facilitate the success of the heterologous expression by targeting multiple synthetic biology levels, including individual proteins, pathways, metabolic flux and hosts. This review describes recent advances in heterologous production of RiPPs, mainly from microbes, with a focus on *E. coli* and *Streptomyces* strains as the surrogate hosts.

## Introduction

**Figure 1:**
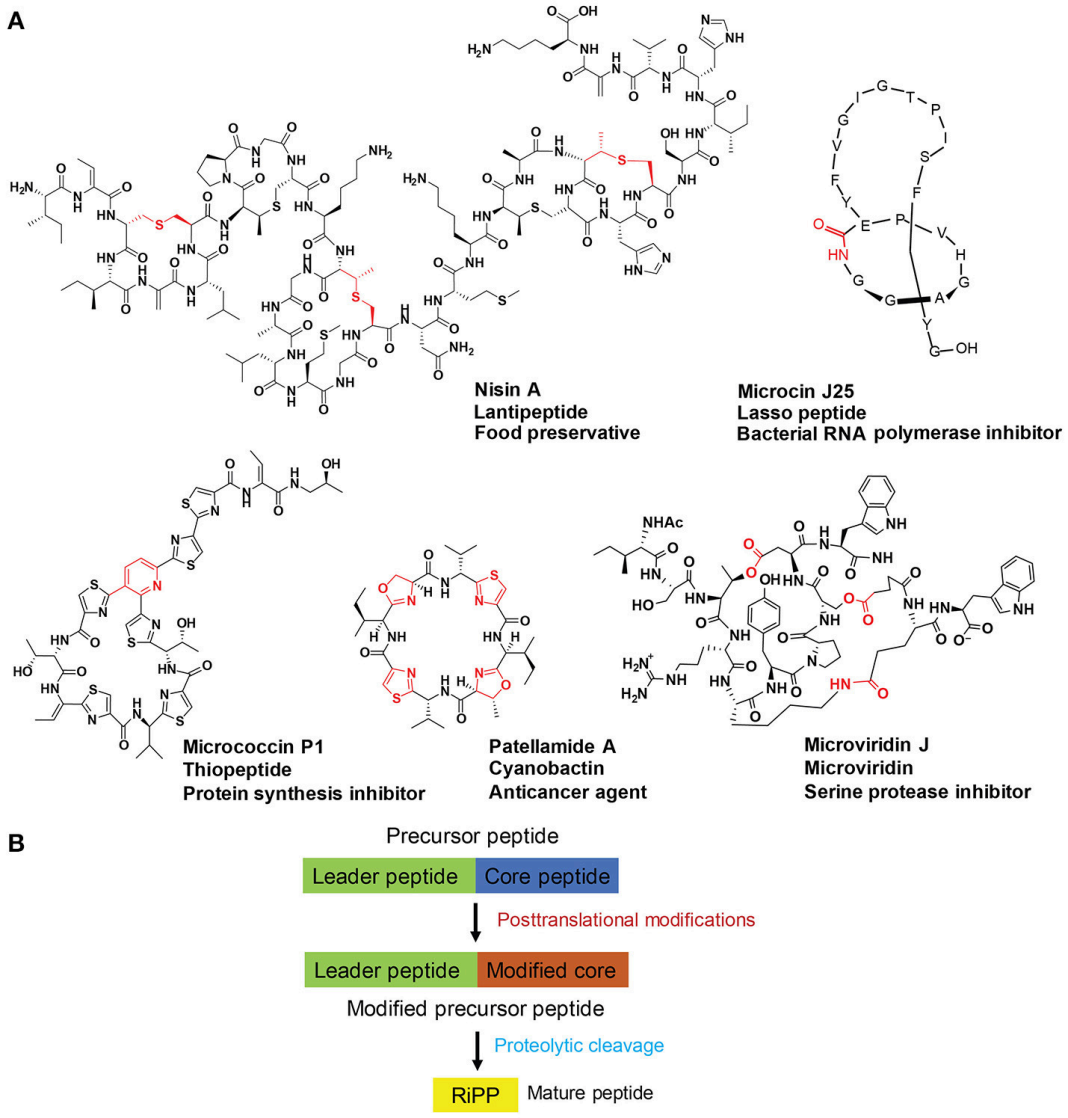
**(A)** Representative structures of five select RiPP families with diverse bioactivities. Post-translational modification(s) on each structure are highlighted in red. **(B)** A schematic depiction of RiPP biosynthesis. Precursor peptide typically contains the leader peptide (in green) followed by the core peptide (in blue). Modifications of the core peptides (in brown) are guided by the leader peptides that interact with processing enzymes. Proteolytic release of the leader peptides then gives rise to mature RiPPs (in yellow).

Ribosomally synthesized and post-translationally modified peptides (RiPPs) are a large group of natural products with a high degree of structural diversity and a wide variety of bioactivities (Figure 1A; Arnison et al., 2013). So far, over 20 different families of RiPPs have been discovered, each carrying unique chemical features (Ortega and van Der Donk, 2016). A biosynthetic logic for RiPPs has emerged and can be simplified as the post-translational modification (PTM) of ribosomally synthesized precursor peptides (Figure 1B; Arnison et al., 2013). An ever-growing list of PTMs expand chemical functionality and often impart metabolic and chemical stability upon precursor peptides. One precursor peptide usually contains the leader peptide (in rare cases *C*-terminal, named as follower peptide) *N*-terminal to the core peptide. The leader peptide binds to and guides biosynthetic enzymes for PTMs on the core peptide and is eventually removed from the modified core peptides by proteases. The entire sequence of the core peptide is generally retained in the final structures of RiPPs and can carry multiple variable sites. As such, the separation of substrate recognition and catalysis enables a concise RiPP biosynthetic route, possessing an evolutionary advantage of accessing high chemical diversity at low genetic cost.

**Figure 2:**
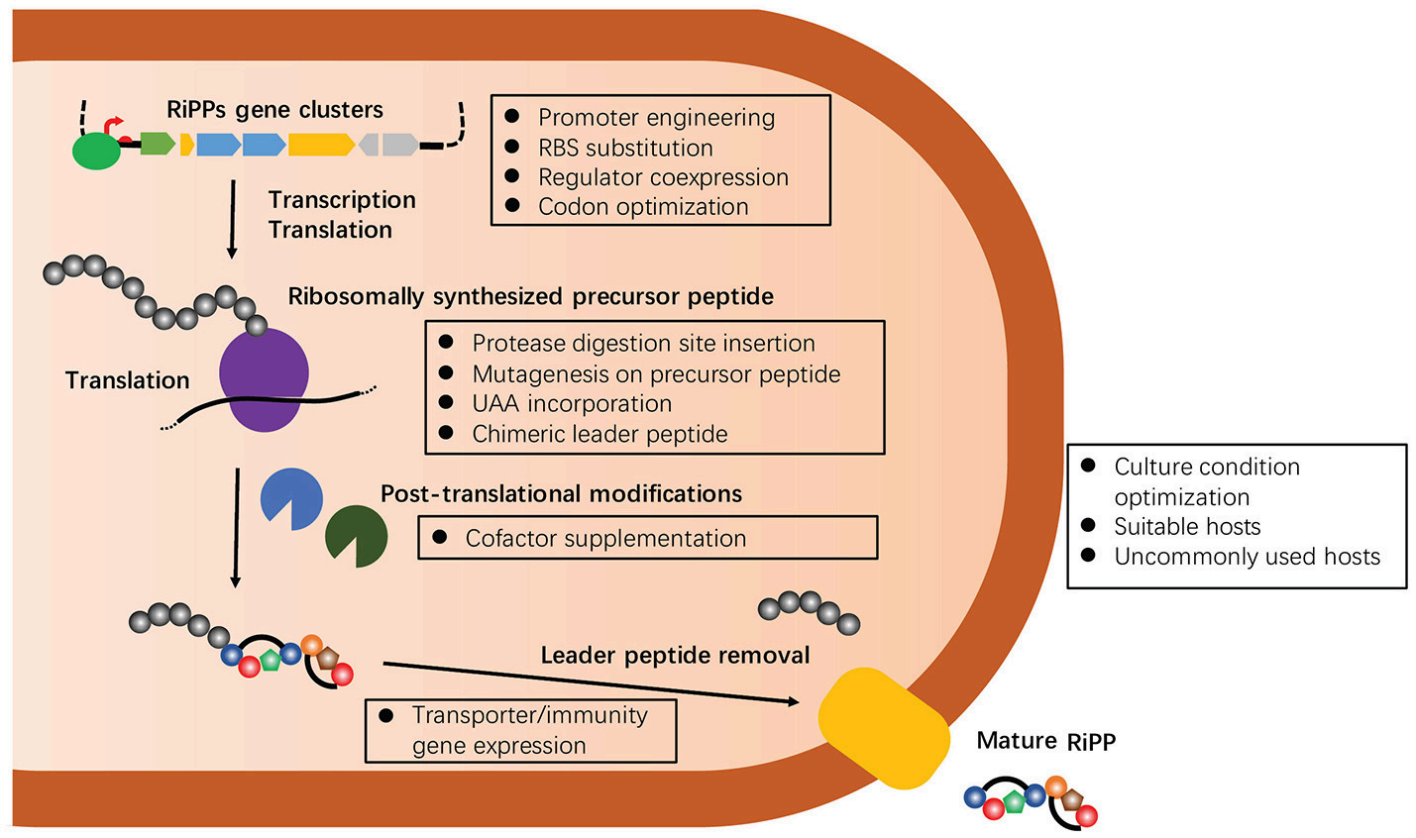
A summary of multiple emerging strategies that target on manipulating individual proteins, pathways, metabolic flux or hosts to improve the success of heterologous expression of RiPPs. All of these strategies will be discussed below with select recent examples.

As a consequence of their ribosomal origin, the chemical structures of RiPPs are more predictable from genomic data than other families of natural products, making RiPPs an attractive target of genome-driven natural product discovery efforts. Compared to conventional “top-down” approaches, the starting point of the genome-driven approach is genome sequences that have exponentially grown over the past decade. Many specialized bioinformatic tools have been developed for identifying RiPPs biosynthetic gene clusters, such as AntiSMASH (Weber et al., 2015), PRISM (Skinnider et al., 2017), SMURF (Khaldi et al., 2010), and more recently RODEO (Tietz et al., 2017). However, there are many technical challenges to translate the identified clusters into chemical entities, rendering the genome-driven approach far from being a panacea for accessing the chemical space that natural products occupy (Luo et al., 2014). Indeed, the diversity and complexity of PTMs, which are often essential for bioactivity of RiPPs, are not readily identifiable on the core peptides as our understanding of biosynthetic enzymes, particularly their substrate specificity and regio-, stereo-, and chemo-selectivity, remains limited (Arnison et al., 2013). On the other hand, the structural determination of RiPPs is often challenged with their no-to-low isolation yields from samples collected from the field or cultured under laboratory conditions (Smith et al., 2018). Over the past decade, many approaches have been developed to address this critical, major issue of the genome-driven approach, including the activation of silent biosynthetic gene clusters (e.g., modification of fermentation methods and engineering of original producers), heterologous expression using a genetically tractable surrogate host, and *in vitro* reconstruction (Chiang et al., 2011; Abdelmohsen et al., 2015; Reen et al., 2015; Ren et al., 2017). Among them, heterologous expression of RiPPs in surrogate hosts, commonly *Escherichia coli* and *Streptomyces* strains, has so far been one of the most successful methods to elucidate cryptic gene clusters and discover new RiPPs (Ortega and van Der Donk, 2016). Furthermore, heterologous production can effectively harvest the promiscuity of RiPP biosynthetic systems to produce designed analogs through genetic engineering of precursor peptides. Importantly, many emerging strategies have been developed to improve the success of heterologous production of RiPPs over the past several years, mainly focusing on the manipulation of individual proteins, pathways, metabolic flux and hosts (Figure 2). Herein, this review describes the details of these strategies ensuring and expanding the heterologous expression approach to discover and develop RiPPs. Representative examples of heterologous expression of each major family of RiPPs were summarized in Table 1. Of note, thousands of antimicrobial peptides have been isolated from a variety of organisms (Deng et al., 2017), and this manuscript excluded their heterologous production in discussions.

**Table 1:** Selected successful examples of heterologous expression of different RiPP families^a^.

**Subfamily of RiPPs**	**Natural products**	**Native host**	**Heterologous host**
Bottromycin	Bottromycin (Huo et al., 2012)	*Streptomyces bottropensis*	*S. coelicolor* A3(2)
Bacteriocin	Bacteriocin enterocin A (EntA) (Jiménez et al., 2015)	*Enterococcus faecium*	*Lactobacillus* spp.
Cyanobactin	Patellamide A and C (Schmidt et al., 2005)	*Prochloron didemni*	*E. coli* BL21 (DE3)
Cyanobactin	Patellamide (Long et al., 2005)	*Prochloron didemni*	*E. coli* DH10B
Cyanobactin	Patellamide and ulithiacyclamide (Donia et al., 2006)	*Prochloron* spp.	*E. coli* Rosetta2 (DE3)
Cyanobactin	Trunkamide (Donia et al., 2008)	*Prochloron* spp.	*E. coli* TOP10
Cyanobactin	Anacyclamides (Leikoski et al., 2010)	*Anabaena* sp. 90	*E. coli* One Shot TOP10
Cyanobactin	Hexameric patellin (Tianero et al., 2012)	*Lissoclinum* sp.	*E. coli* TOP10
Cyanobactin	Trunkamide derivatives (Ruffner et al., 2015)	*Lissoclinum* sp.	*E. coli* 10*-β*
Cyanobactin	Telomestatin (Amagai et al., 2017)	*Streptomyces anulatus* 3533-SV4	*S. avermitilis* SUKA22
Cyclotide	Kalata B1 (Poon et al., 2018)	*Oldenlandia affinis*	*Nicotiana benthamiana*
Lanthipeptide I	Cinnamycin (Widdick et al., 2003)	*Streptomyces cinnamoneus* DSM 40005	*S. lividans* 1326
Lanthipeptide I	Microbisporicin (Foulston and Bibb, 2010)	*Microbispora corallina*	*Nonomuraea* sp. ATCC 39727
Lanthipeptide I	Geobacillin I (Garg et al., 2012)	*Geobacillus thermodenitrificans*	*E. coli* BL21 Gold
Lanthipeptide I	Modified gallidermin and nisin (Van Heel et al., 2013)	*Lactococcus lactis*	*L. lactis* NZ9000
Lanthipeptide I	Planosporicin (Sherwood et al., 2013)	*Planomonospora alba*	*Nonomuraea* sp. ATCC 39727
Lanthipeptide I	NAI-107 (Microbisporicin A1) (Ortega et al., 2016)	*Lactococcus lactis*.	*E. coli* BL21 Gold
Lanthipeptide II	Nukacin ISK-1 (Aso et al., 2004)	*Staphylococcus warneri* ISK-1.	*Lactococcus lactis* NZ9000
Lanthipeptide II	Prochlorosin 1.7, 2.11, 3.2, and 3.3 nisin (Shi et al., 2011)	*Prochlorococcus*	*E. coli* BL21 Gold
Lanthipeptide II	Cinnamycin (Ökesli et al., 2011)	*Streptomyces cinnamoneus* DSM 40005	*E. coli* BL21 Gold
Lanthipeptide II	Lichenicidin (Caetano et al., 2011a)	*Bacillus licheniformis*	*E. coli* BL21 Gold
Lanthipeptide II	Lichenicidin (Caetano et al., 2011b)	*Bacillus licheniformis*	*E. coli* BL21 Gold
Lanthipeptide II	Prochlorosin analogs (Tang and Van Der Donk, 2012)	*Prochlorococcus* MIT9313	*E. coli* BL21 Gold
Lanthipeptide II	Carnolysin (Lohans et al., 2014)	*Carnobacterium maltaromaticum* C2	*E. coli* BL21 Gold
Lanthipeptide II	Bovicin HJ50-like lantibiotics (Wang et al., 2014)	*Streptococcus bovis* HJ50	*E. coli* BL21 Gold
Lanthipeptide II	Lichenicidin (Kuthning et al., 2015)	*Bacillus licheniformis* I89	*E. coli* BL21 Gold
Lanthipeptide II	Pseudomycoicidin (Basi-Chipalu et al., 2015)	*Bacillus pseudomycoides*	*E. coli* C43
Lanthipeptide II	Lanthipeptides (Zhao and Van Der Donk, 2016)	*Ruminococcus flavefaciens*	*E. coli* BL21 Gold
Lanthipeptide IV	Streptocollin (Iftime et al., 2015)	*Streptomyces collinus* Tì 365	*S. coelicolor* M1146 and M1152
Lasso peptide	Capistruin (Knappe et al., 2008)	*Burkholderia thailandensis* E264	*E. coli* BL21 Gold
Lasso peptide	Microcin J25 (Pan and Link, 2011)	*E. coli* AY25	*E. coli* XL-1 Blue
Lasso peptide	Astexin-1 (Maksimov et al., 2012)	*Asticcacaulis excentricus*	*E. coli* BL21 Gold
Lasso peptide	Astexin-2 and−3 (Maksimov and Link, 2013)	*Asticcacaulis excentricus*	*E. coli* BL21 Gold
Lasso peptide	Burhizin, Caulonodin I, Caulonodin II, Caulonodin III, Rhodanodin, Rubrivinodin, Sphingonodin I, Sphingonodin II, Syanodin I, Sphingopyxin I, Sphingopyxin II, and Zucinodin (Hegemann et al., 2013b)	Multiple proteobacterial strains	*E. coli* BL21 Gold
Lasso peptide	Caulonodins IV to VII (Zimmermann et al., 2014)	*Caulobacter* sp. K31	*E. coli* BL21 Gold
Lasso peptide	MccJ25 UAA (Piscotta et al., 2015)	*E. coli* AY25	*E. coli* BL21 Gold
Lasso peptide	Benenodin-1 and−2 (Chekan et al., 2016)	*Asticcaucalis benevestitus*	*E. coli* BL21 Gold
Linaridin	Grisemycin (Claesen and Bibb, 2011)	*Streptomyces griseus* IFO 13350	*S. coelicolor* M1146
Microviridin	Microviridin J (Ziemert et al., 2008)	*Microcystis* UOWOCC MRC	*E. coli* Epi300
Microviridin	Microviridin L (Weiz et al., 2011)	*M. aeruginosa* NIES843	*E. coli* BL21
Omphalotin	Omphalotin A (Ramm et al., 2017)	*Omphalotus olearius*	*Pichia pastoris* GS115
Sactipeptides	Subtilosin A (Himes et al., 2016)	*B. subtilis* 168	*E. coli* BL21 (DE3)
Thiopeptide	Thiazolyl peptide GE37468 (Young and Walsh, 2011)	*Streptomyces* ATCC 55365	*S. lividans* TK24
Thiopeptide	Thiopeptide GE2270 (Tocchetti et al., 2013)	*Planobispora rosea*	*Nonomuraea* ATCC39727
Thiopeptide	Berninamycin (Malcolmson et al., 2013)	*Streptomyces bernensis* UC 5144	*S. lividans* TK24, *S. venezuelae* ATCC 10712
Thiopeptide	Silent thiopeptide biosynthetic Lactazoles gene cluster (Hayashi et al., 2014)	*Streptomyces lactacystinaeus* OM-6519	*S. lividans* TK23
Thiopeptide	Thiopeptide antibiotic GE2270 (Flinspach et al., 2014)	*Planobispora rosea* ATCC 53733	*S. coelicolor* M1146
Thioviridamide	Thioviridamide (Izawa et al., 2013)	*Streptomyces olivoviridis*	*S. lividans* TK23
Thioviridamide	JBIR-140 (Izumikawa et al., 2015)	*S. olivoviridis* OM13	*S. avermitilis* SUKA17
TOMM	Plantazolicin (Deane et al., 2013)	*Bacillus amyloliquefaciens* FZB42	*E. coli* BL21 (DE3)
TOMM	Microcin B (Metelev et al., 2013)	*Pseudomonas syringae*	*E. coli* BL21 (DE3)
Ustiloxin	Ustiloxin B (Ye et al., 2016)	*Ustilaginoidea virens*	*Aspergillus oryzae*

a *Entries were arranged first by the alphabetical order of the names of RiPP families and then chronically by the year of the publication*.

## Manipulation of components of RiPP biosynthetic pathways

A RiPP gene cluster commonly comprises of all essential genes for the production of RiPP. Manipulation of the pathway-specific components allows precise and rational improvement of RiPP production and minimizes potential perturbation of the holistic metabolism of the heterologous host. Detailed information regarding the function, timing, specificity and regulation on the pathway can also be extracted via this approach. From a synthetic biology standpoint, here we use representative examples to describe different strategies used to manipulate RiPP biosynthetic pathways for successful heterologous expression.

### Promoter engineering to control gene transcription

**Figure 3:**
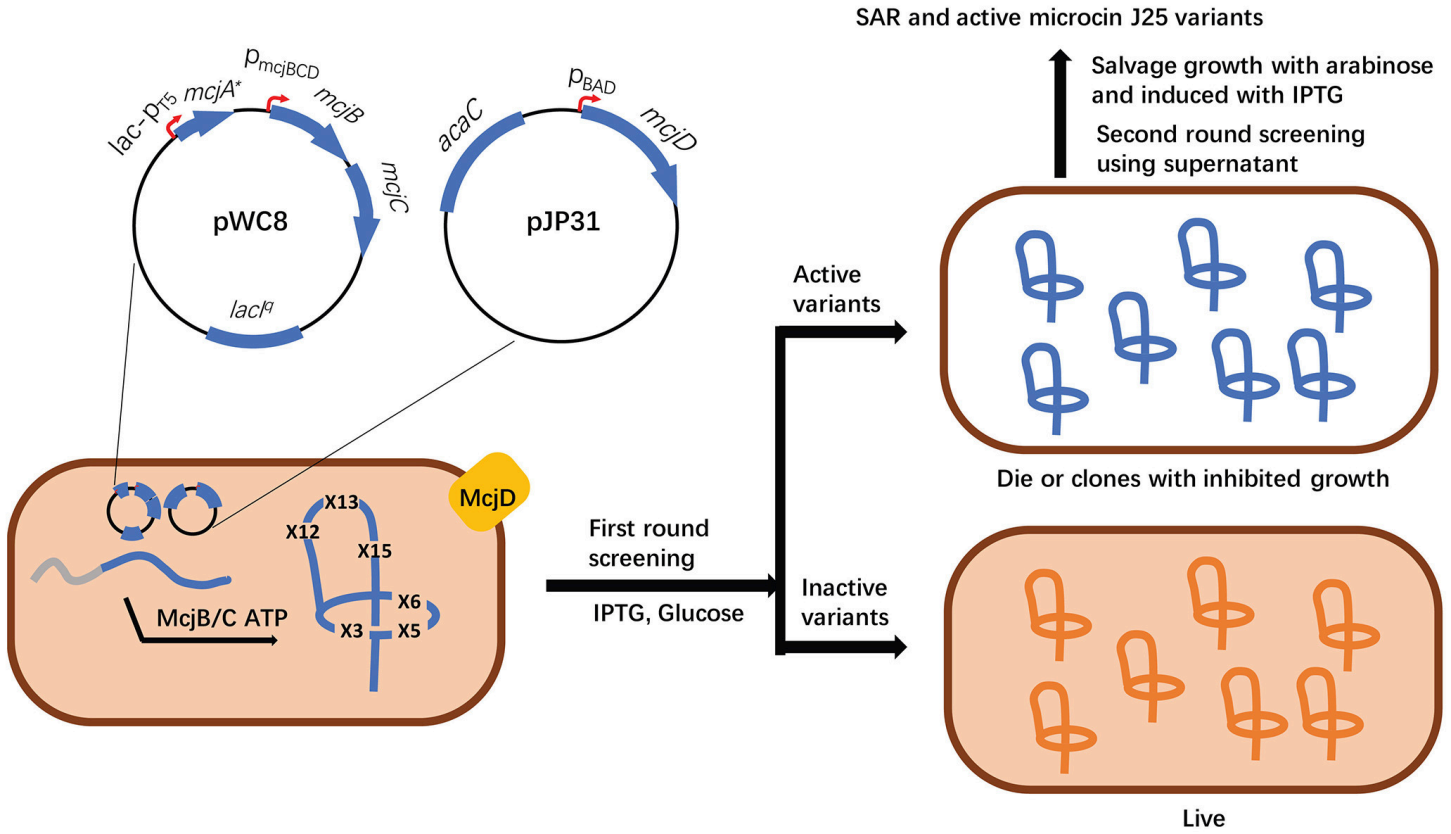
High throughput discovery of functional microcin J25 variants with multiple amino acid substitutions was enabled by an orthogonally inducible system which separately controls the production and export/immunity of mature RiPPs. More specifically, the expression of the precursor gene *mcjA* and the transporter gene *mcjD* was independently induced by IPTG and arabinose, respectively. In the noninduced state, leaky expression leads to the low levels of both McjA and McjD (left). When IPTG and glucose are added, the expression of *mcjA* mutants is highly induced, but not *mcjD*, resulting in cytoplasmic accumulation of McjAs. If McjAs are processed into mature MccJ25 variants with antibacterial activity, accumulated lasso peptides will inhibit the growth of the host cell (top right). The poor growth of these cells will be salvaged by the addition of arabinose to overexpress McjD. By contrast, inactive MccJ25 variants will have no inhibitory effect on the cell growth (bottom right).

Altered transcription levels of biosynthetic genes are commonly observed when they are introduced into heterologous hosts. Genetic engineering of a biosynthetic gene cluster by the introduction of one or more constitutive or inducible promoters has proved very effective for the heterologous production of different RiPP families. Importantly, a number of well-characterized promoters of commonly used hosts (e.g., *E. coli* and *Streptomyces* strains) (De Mey et al., 2007; Li et al., 2015; Myronovskyi and Luzhetskyy, 2016) have been available to enable this synthetic biology approach. For example, lichenicidin is a two-component lantibiotic produced by *Bacillus licheniformis* I89, and its heterologous production from the native gene cluster in *E. coli* BLic5 led to a significantly lowered yield compared with the native producer (Table 1; Caetano et al., 2011a,b). By contrast, driving the expression of each biosynthetic gene by a strong T7 promoter resulted in a yield of lichenicidin up to 100 times higher than *B. licheniformis* I89 (Kuthning et al., 2015). In another example, *Staphylococcus warneri* ISK-1 produces a lantibiotic nukacin ISK-1 (Sashihara et al., 2000) but the heterologous expression of its gene cluster in *S. carnosus* TM300 and *Lactobacillus plantarum* ATCC 14917^T^ failed to produce any natural product (Aso et al., 2004). Aso et al. addressed this problem through the identification of a cognate response activator and by driving the cluster expression with a nisin-inducible promoter PnisA (Table 1; Aso et al., 2004). Likewise, the utilization of a proper promoter was also essential for the successful production of a macrocyclic peptide telomestatin (Table 1). Initially, a xylose-inducible promoter (xylAp) was used to drive the expression of its gene cluster in the highly engineered *Streptomyces avermitilis* SUKA17 (Komatsu et al., 2013) but yielded no targeted molecule. It was later speculated that the transcription of the gene cluster should be activated during the late logarithmic phase of cell growth. Accordingly, the replacement of xylAp with the olmRp promoter led to the production of telomestatin in *S. avermitilis* SUKA17 (Amagai et al., 2017), clearly indicating the essentiality and importance of temporal control of gene expression in the successful production of natural products. Other remarkable examples of applying constitutive or inducible promoters to promote the success of RiPP heterologous expression include the complete refactoring of the cyanobactin patellamide pathway for its expression in *E. coli* Rosetta2 (DE3) (Donia et al., 2006), the use of inducible araP_BAD_ promoter to drive the entire operon of a lasso peptide in *E. coli* BL21 (DE3) (Metelev et al., 2013), increased production of thiopeptides GE2270 and lactazole A in *Streptomyces* hosts after introduction of the constitutive ermE^*^ promoter (Flinspach et al., 2014) and by a strong promoter (Hayashi et al., 2014), respectively (Table 1). Of note, the Link group constructed an expression system with two orthogonally inducible promoters to permit a separate control of the production and the export/immunity of lasso peptide MccJ25 in *E. coli* (Table 1, Figure 3). This elegant design enabled high-throughput screening of saturation mutagenesis libraries of the ring and β-hairpin tail regions of MccJ25 to obtain new insights to its structure-activity relationship (Pan and Link, 2011).

### RBS substitution to optimize translation efficiency

A ribosomal binding site (RBS) is critical in initiating the translation of many downstream genes. Its efficiency depends on the core Shine-Dalgarno (SD) sequence, the surrounding secondary structure, and the spacing between the SD sequence and the start codon AUG. Upon translation initiation, the 3′-sequence of the 16S rRNA complementarily pairs with the SD sequence in the RBS. Over millions of years of evolution, microbes have created and utilized a diverse set of RBSs to control protein translation (Omotajo et al., 2015), which is also employed to regulate the production of secondary metabolites. As such, RBSs are an important component part of synthetic biology applications including the heterologous production of RiPPs and other families of natural products (Bai et al., 2015). For example, the incorporation of optimized *E. coli* RBSs has proven to be an efficient way to significantly increase the yields of multiple lasso peptides, including astexin–1,−2, and−3 (Maksimov et al., 2012; Maksimov and Link, 2013), capistruin (Pan et al., 2012), and caulosegnin (Table 1) (Hegemann et al., 2013a). In a more inclusive example, Hegemann et al. cloned the gene clusters of lasso peptides from various sources into the expression vector pET41a, and included a strong *E. coli* RBS in the intergenic region between their precursor gene(s) and the genes encoding processing enzymes (Table 1; Hegemann et al., 2013b). This design increased the production yields of almost all expressed lasso peptides by 1.8- to 84.5-folds, although the deletion of extra precursor peptides might also contribute to the yield improvement in some cases (Hegemann et al., 2013b).

### Optimization of the catalytic performance of processing enzymes

RiPP biosynthesis recruits a rapidly expanding list of functionally diverse enzymes to furnish structural and functional diversity (Arnison et al., 2013). The reactions of some RiPP biosynthetic enzymes require cofactors/co-substrates that may not be (or insufficiently) available in the surrogate host, leading to suboptimal production of targeted RiPPs. Therefore, optimal heterologous expression of RiPPs sometimes can be achieved by targeting cofactors/co-substrates of essential processing enzymes. For instance, NisB is a dehydratase involved in the biosynthesis of the food preservative nisin and its catalytic function requires glutamyl-tRNA^Glu^ as a co-substrate, uncommon to RiPP processing enzymes (Ortega et al., 2016). Accordingly, increasing the cellular availability of *Microbispora* sp. 107891 glutamyl-tRNA^Glu^ in *E. coli* was attempted to enhance the catalytic activity of MibB, a homolog of NisB involved in the biosynthesis of NAI-107. This study led to the production of NAI-107 analogs containing up to seven dehydrations, in contrast to nearly no dehydration when having no expressed *Microbispora* sp. 107891 glutamyl-tRNA^Glu^ (Table 1) (Ortega et al., 2016). In a more pronounced example, the Schmidt group found that the addition of cysteine (5–10 mM) to the culture media, along with minor process changes, increased the yield of cyanobactin patellins by 150-folds (Table 1; Tianero et al., 2016). It was proposed that sulfide derived from cysteine specifically modulates the substrate preference of cyanobactin processing enzymes, enabling post-translational control of product formation *in vivo*. Moreover, elevating the availability of the isoprene precursor, which is required by the pathway-specific prenyltransferase (Mcintosh et al., 2011), gave rise to an additional ~18-fold increase of patellin yield in *E. coli* (Table 1).

### Codon optimization to enhance heterologous expression

Due to the different abundance of tRNAs in various hosts, each organism has its own codon preference. Thus, codon optimization of biosynthetic genes proves to be a good strategy to achieve optimal heterologous expression. For example, the biosynthetic genes of geobacillin I, a nisin analog encoded by the thermophilic bacterium *Geobacillus thermodenitrificans* NG80-2, were codon-optimized before their introduction to *E. coli* for heterologous expression (Garg et al., 2012). Likewise, genes *cylL*_*L*_, *cylL*_*S*_, and *cylM* encoding the enterococcal cytolysin were synthesized with codon optimization for use in *E. coli* (Tang and Van Der Donk, 2013). Notably, in the heterologous expression of patellamides in *E. coli*, much lower yield was observed with vectors that were not codon-optimized (Schmidt et al., 2005).

### Manipulation of pathway-specific regulators

Despite the brevity of RiPP biosynthetic logic (Figure 1B), their gene clusters often encode components for precursor peptides, processing enzymes, resistance mechanism and regulators, the same as other families of natural products (e.g., polyketides and nonribosomal peptides) (Ortega and van Der Donk, 2016). Targeting any of these components, particularly the regulators of RiPP biosynthetic pathways, can favor the success of RiPP heterologous production. A comprehensive review on gene-regulatory mechanisms operating in RiPPs biosynthesis was recently reported elsewhere (Bartholomae et al., 2017). We highlighted here an example about the essentiality of a pathway-specific regulator to successful RiPP heterologous expression. The biosynthetic gene cluster of thiopeptide GE2270 (pbt) from *Planobispora rosea* ATCC 53733 previously failed to express the natural product in several *Streptomyces* hosts (Table 1; Tocchetti et al., 2013). In a recent report, Flinspach et al. revealed that the expression of PbtR, a TetR family of transcriptional regulator, is essential to the successful heterologous production in *S. coelicolor* M1146 (Flinspach et al., 2014).

### Engineering resistance mechanisms to improve RiPP productivity

Natural products are known to possess biological activities that target organisms in the same environmental niches, thereby offering survival benefits (Behie et al., 2017). To avoid self-toxicity, the producers accordingly evolve many different types of resistance mechanisms (e.g., transporters, chemical modification and target modification), often embedded in the natural product gene clusters (Jia et al., 2017; Almabruk et al., 2018). Expectedly, resistance mechanisms can offer a way to regulate the production of natural products, including RiPPs. For example, the biosynthesis of the lantibiotic nisin in *Lactococcus lactis* requires the dehydratase NisB, the cyclase NisC, the ABC-type transporter NisT, and the protease NisP, which together convert the precursor peptide NisA into the final product (Cheigh and Pyun, 2005). NisT forms a protein complex with NisB, C and P to effectively export bioactive nisin after its formation. Indeed, no secreted nisin was detected from the medium of a *L. lactis* mutant lacking the *nisT* gene, while the expression of *nisABCP* in this strain resulted in a considerable growth inhibition due to the intracellular accumulation of nisin (Table 1; Van Den Berg Van Saparoea et al., 2008). This example illustrates the necessity of a resistance mechanism to protect RiPP native producers. The same is likely true to surrogate hosts. For example, the ABC transporter MdnE was reported to be crucial for the successful production of a unique RiPP family, cyanobacterial tricyclic microviridins, in *E. coli* (Table 1; Weiz et al., 2011). In this case, MdnE might also act as a scaffold protein to guide the biosynthesis (Weiz et al., 2011). In another example, the multidrug transporter BotT of a bottromycin biosynthetic pathway is key to produce this antibiotic peptide in the surrogate host (Huo et al., 2012). Overexpression of the *botT* gene driven by a strong PermE^*^ promoter in *S. coelicolor* host enhanced the production titer by 20 times compared to the control with the unmodified cluster. In addition to transporter genes, other resistance-imparting genes can also be used to boost the heterologous production of RiPPs. For instance, the heterologous production of the bacteriocin enterocin A (EntA) was accomplished by fusing a Sec-dependent signal peptide (SPusp45) with mature EntA and coexpressing the EntA immunity gene *entiA* (Table 1; Jiménez et al., 2015). EntiA protects the producing strain by forming a strong complex with the receptor protein, mannose phosphotransferase system, to avoid the toxicity. These manipulations led to a 4.9-fold higher production of EntA than the native producer (Jiménez et al., 2015).

### Engineering precursor peptides to produce RiPPs and their analogs

For the successful heterologous expression of RiPPs, one common hurdle is the lack of proper peptidases in the surrogate host to remove the leader peptide after finishing modifications on the core peptide (Bindman et al., 2015). Indeed, a number of RiPP gene clusters do not encode a protease dedicated to the removal of the leader peptide. The sequences of the linkers between the leader and core peptides also provide limiting information for the identification of such a protease from the genomes of native producers. To address this issue, the digestion site of a well-characterized, commercially available protease, such as GluC (Tang and Van Der Donk, 2012; Zhao and Van Der Donk, 2016;), trypsin (Himes et al., 2016), and C39 protease domain of the ABC transporter (Wang et al., 2014), can be engineered into the linker for the *in vitro* proteolytic release of the leader peptide from the matured precursor peptides isolated from heterologous hosts. As another approach, the van der Donk group genetically incorporated unnatural amino acids (UAAs) hydroxyl acids in the first position of a lanthipeptide by using a pyrrolysyl-tRNA synthetase-tRNA${}_{\text{CUA}}^{\text{Pyl}}$ pair in *E. coli* (Table 1; Bindman et al., 2015). The installation of hydroxyl acid leads to an ester linkage between the leader and core peptides, which is readily cleavable by simple hydrolysis.

The majority of RiPPs precursor peptides comprise of the leader peptide region for the interactions with processing enzymes and the core peptide region that becomes the final products after chemical modification and proteolytic removal of the leader peptide (Arnison et al., 2013). The core region often carries multiple sequence variations that are tolerated by processing enzymes in modifications, providing opportunities to expand the chemical diversity of RiPPs. Indeed, genetic engineering of the core peptides of multiple RiPP families has led to impressive successes in exploring new chemical space for therapeutic applications. Two strategies have commonly been employed to diversify core peptide sequences, including single-site saturation mutagenesis (Young et al., 2012) and multiple-site sequence randomization (Ruffner et al., 2015; Yang et al., 2018). The first strategy is advantageous to screen small-size libraries but can miss desirable mutants that require multiple mutations on the core peptides. By contrast, the second strategy in principle explores the broadest chemical space covered by large libraries (e.g., 10^6^-10^9^ members), which is favored in drug discovery and development research. However, the success of this strategy depends on all three following factors, (1) the expression of all precursor peptide mutants in the host, (2) the proper processing of all mutants to generate large numbers of RiPP analogs, and (3) the high throughput screening methods to identify desirable compounds. In one recent example, Ruffner et al. employed the second strategy to randomly mutate the core peptide (TSIAPFC) of cyanobactin trunkamide (Table 1; Ruffner et al., 2015), whose processing enzymes are known to exhibit unusually relaxed sequence selectivity (Sardar et al., 2015). They prepared three double mutant libraries (XXIAPFC, TSXXPFC, and TSIXPXC) and a quadruple mutant library (XSXXPXC) in *E. coli* using the degenerate codon NNK. From the double mutant libraries (theoretically, 1,200 unique sequences in each library), they randomly screened a total of 460 clones, found 260 full-length precursor peptides, and detected 150 trunkamide analogs, giving a 33% success rate. The quadruple mutant library had the potential to produce 160,000 different sequences. The authors assessed the quality of this library by screening randomly picked 96 clones, found 65 full-length precursor peptides, and detected nine trunkamide analogs. The lower success rate (9.4%) of the quadruple mutant library may correlate with the selectivity of processing enzymes. In this regard, the van der Donk group recently leveraged the remarkable substrate tolerance of a lanthipeptide synthetase ProcM to generate a genetically encoded lanthipeptide library (Yang et al., 2018). They first randomized 10 positions of the core peptide of the precursor peptide ProcA2.8 (Table 1) (AACXXXXXSMPPSXXXXXC) using the NWY codon that encodes eight amino acids, leading to a 1.07 × 10^9^ library. Limited by the transformation efficiency of *E. coli*, they obtained ~10^6^ clones, 99.7% of which produced unique peptide sequences. Screening of 33 randomly selected clones led to identify 33 cyclized samples, illustrating the impressive versatility and substrate flexibility of ProcM. The authors then screened all 10^6^ lanthipeptides using a cell survival-based high throughput assay and identified one potent inhibitor of HIV p6 protein (Yang et al., 2018). In addition to the use of *E. coli* as a host to produce mutated RiPPs, both yeast display and phage display have recently been used to generate libraries of 10^6^ lanthipeptides for screening for new bioactive analogs (Urban et al., 2017; Hetrick et al., 2018). These two well-characterized platforms can find more applications in expanding the chemical space of other RiPP families by the sequence randomization strategy.

In addition to 20 proteinogenic amino acids, a variety of UAAs can be used to expand the chemical diversity of RiPPs (Young and Schultz, 2010). This strategy has demonstrated its success with multiple RiPP families, including lantipeptide (Nagao et al., 2005; Oldach et al., 2012; Bindman et al., 2015; Kuthning et al., 2016; Lopatniuk et al., 2017; Zambaldo et al., 2017), lasso peptide (Piscotta et al., 2015), cyanobactin (Tianero et al., 2012), and sactipeptide (Himes et al., 2016). However, these unnatural RiPP analogs showed no significant improvement of their bioactivities possibly due to the relatively small extent of chemical expansion brought by a single UAA on a single position. However, coupled with the directed evolution of targeted core peptides, e.g., multiple-site randomization as described above, this strategy can generate new-to-nature RiPP analogs with enhanced structural and functional diversity.

**Figure 4:**
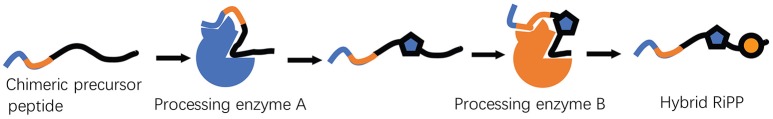
A chimeric leader peptide strategy to produce unnatural RiPP hybrids. By properly designing the concatenated leader peptides, recognition and processing by multiple enzymes from unrelated RiPP pathways could be realized. By using this method, a thiazoline-forming cyclodehydratase was combined with biosynthetic enzymes from the sactipeptide and lanthipeptide families to create new-to-nature hybrid RiPPs, demonstrating the feasibility of the strategy.

RiPP precursor peptides physically separate their molecular recognition and catalysis sites for the processing by enzymes. Capitalizing on this distinct feature, a chimeric leader peptide strategy was recently developed to produce RiPP hybrids (Burkhart et al., 2017). Specifically, the leader peptides for the binding of thiazoline-forming cyclodehydratase, thioether-formation AlbA involved in the biosynthesis of sactipeptide, and lanthipeptide dehydratases NisB/C and ProcM were fused to allow sequential interactions with multiple processing enzymes of different RiPP families (Figure 4). As such, the engineered core peptides were received a combination of chemical transformations to produce unnatural peptide products, providing a generally applicable strategy to unlock the vast chemical space afforded by a variety of RiPP biosynthetic machinery (Burkhart et al., 2017).

## Manipulation of surrogate hosts for the production of RiPPs

### Optimization of culture conditions

Screening a wide array of fermentation conditions, e.g., temperature, pH, shaking speed, nutrient levels, and trace metals, has routinely been practiced for the optimal production of target products. For example, Knappe et al. heterologously expressed the gene cluster of lasso peptide capistruin in *E. coli* and achieved a yield of 0.2 mg/L in the defined medium M20, which was 30% of its native producer *Burkholderia thailandensis* E264 in the same medium (Table 1) (Knappe et al., 2008). Surprisingly, no capistruin was produced when culturing transformed *E. coli* in commonly used LB medium. In another example, after testing a variety of conditions, the co-expression of Fe-S cluster biogenesis genes and lowered shaking speed together led to the significantly improved expression of subtilosin A in *E. coli* (Himes et al., 2016).

### The use of suitable hosts for the production of RiPPs

**Figure 5:**
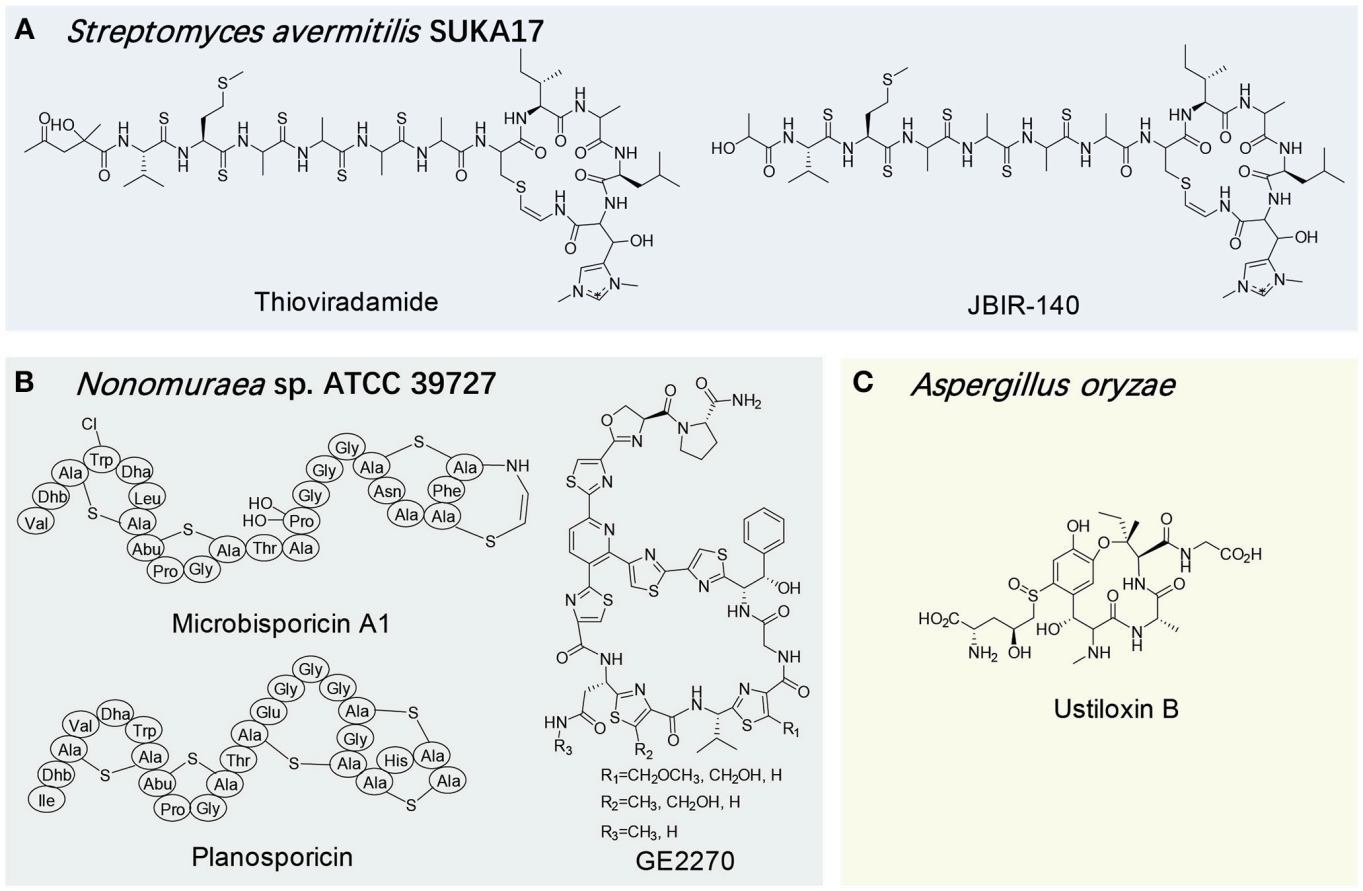
Structures of select RiPPs produced by uncommon surrogate hosts exemplified by *Streptomyces avermitilis* SUKA17 **(A)**, *Nonomuraea sp*. ATCC 39727 **(B)** and *Aspergillus oryzae* **(C)**.

An ideal host for the heterologous expression of natural products usually requires a clean background and high compatibility with the target biosynthetic gene cluster. More specifically, the ideal heterologous host would be able to supply abundant biosynthetic precursors from its primary metabolism while maintaining a relative clean secondary metabolic background, and also be capable of recognizing exogenous genetic parts, thus allowing access to the vast biosynthetic potential of the host. In this regard, *E. coli* has become one of the most popular heterologous hosts, and produced many RiPP families, e.g., cyanobactins, lantipeptides, lasso peptides, microviridins, and sactipeptides (Donia et al., 2006; Weiz et al., 2011; Metelev et al., 2013; Himes et al., 2016; Kuthning et al., 2016). On the other hand, the RiPP gene cluster from a high G+C producer is often expressed in a host with a relatively comparable genetic background. For example, the lantibiotic cinnamycin is produced by several *Streptomyces* strains and its gene cluster from *S. cinnamoneus cinnamoneus* DSM 40005 was successfully expressed in *S. lividans* to produce this peptidic antibiotic (Widdick et al., 2003). In another study, *S. lividans* TK23 and *S. avermitilis* SUKA17 were used as the hosts to produce thioviridamide (Table 1, Figure 5A; Izawa et al., 2013; Izumikawa et al., 2015). Interestingly, the expression of its gene cluster in *S. avermitilis* SUKA17 led to the production of a novel thioviridamide derivative, JBIR-140, further demonstrating the significant influence of a surrogate host on RiPP production.

In addition to the widely used hosts like *E. coli* and *Streptomyces* strains, several uncommon microorganisms have also been characterized as suitable RiPP heterologous hosts. With the consideration of available substrates and comparable genetic backgrounds, these heterologous hosts are often from the same family of the native producers of the target RiPPs. For example, when expressing the clusters of the lantibiotics microbisporicin and planosporicin and the thiopeptide GE2270 from *Microbispora coralline, Planomonospora alba* and *Planobispora rosea*, respectively, *Nonomuraea* sp. ATCC 39727, which is in the same family of the above native producers, acted as a viable host to produce corresponding RiPPs, but not multiple other tested *Streptomyces* strains (Table 1, Figure 5B; Foulston and Bibb, 2010; Sherwood et al., 2013; Tocchetti et al., 2013).

In recent years, fungal RiPPs have attracted increasing attentions given the availability of a number of fungal genomes in public domain (Hallen et al., 2007; Ding et al., 2016; Nagano et al., 2016; Ramm et al., 2017). To realize the chemical and functional potential of fungal RiPPs, their heterologous expression systems have to be established. In this regard, commonly used fungal strains can be initial targets in the development. Encouragingly, several biosynthetic genes of ustiloxin, the first filamentous fungal RiPP, were successfully expressed in *Aspergillus oryzae*, greatly facilitating the understanding of the macrocyclic formation and its entire biosynthetic pathway (Table 1, Figure 5C; Ye et al., 2016). In a more recent example, the partial reconstitution of the biosynthesis of one dodecapeptide omphalotin A, which is ribosomally produced by the basidiomycete *Omphalotus* olearius, was succeeded in *Pichia pastoris* strain GS115, but not *E. coli* (Ramm et al., 2017). This work further shed light on a novel biosynthesis mechanism for a RiPP in which a self-sacrificing enzyme, methyltransferase OphMA, bears its own precursor peptide.

Cyclotides are a family of plant-derived RiPPs that are characterized by a head-to-tail cyclic peptide backbone and a cystine knot arrangement of disulfide bonds. These peptidic compounds possess a wide range of bioactivities (e.g., protease inhibition, anti-microbials, and cytotoxicity) and are good carriers of other bioactive peptides, both of which are attractive to pharmaceutical research. Recently, the heterologous production of cyclotides were successfully achieved by co-expressing a select asparaginyl endoprotease and its precursor peptide *in planta*, using *Nicotiana benthamian*, tobacco, bush bean, lettuce, and canola as hosts (Poon et al., 2018). Interestingly, alternative strategies such as intein-mediated protein trans-splicing (Jagadish et al., 2013) and sortase-induced backbone cyclization (Stanger et al., 2014) have also been developed to produce cyclotides in bacterial and yeast expression systems, in which the asparaginyl endoprotease is not employed for the cyclization.

## Conclusion and future perspectives

Harnessing the biosynthetic prowess of RiPPs via heterologous expression has witnessed several exciting advances in recent years. As described above, due to the conciseness of the biosynthetic route, the cloning and mobilization of the RiPP gene clusters typically do not constitute a major hurdle for the heterologous production of RiPPs. However, the functional expression of biosynthetic genes in surrogate hosts could be complicated by many less-predictable factors, such as the availability of protein cofactors, promoter recognition, product toxicity, protein–protein interaction, and imbalanced protein dosage. On the other hand, with *E. coli* and *Streptomyces* strains serving as the most common hosts in the heterologous expression of RiPPs, the ever-increasing number of synthetic biology tools developed for these systems can be applied to overcome these challenges. In addition, *in vitro* characterization of RiPP biosynthesis and *in silico* prediction can be coupled to streamline and improve the outcomes of heterologous expression efforts. We are optimistic that a small set of highly developed hosts will be available as generally applicable platforms for rapid and robust sampling of the vast chemical space of RiPPs from bacteria, fungi, and even plants in future.

## Author contributions

YZ, MC, SB, and YD planned, wrote and reviewed the manuscript.

### Conflict of interest statement

The authors declare that the research was conducted in the absence of any commercial or financial relationships that could be construed as a potential conflict of interest.
